# A bioinformatics potpourri

**DOI:** 10.1186/s12864-017-4326-x

**Published:** 2018-01-19

**Authors:** Christian Schönbach, Jinyan Li, Lan Ma, Paul Horton, Muhammad Farhan Sjaugi, Shoba Ranganathan

**Affiliations:** 1 International Research Center for Medical Sciences, Graduate School of Medical Sciences, Kumamoto University, Kumamoto, 860-0811 Japan; 2 The Advanced Analytics Institute, University of Technology Sydney, Sydney, NSW 2007 Australia; 3 Graduate School at Shenzhen, Tsinghua University, Shenzhen, 518055 People’s Republic of China; 4 Artificial Intelligence Research Center, National Institute of Advanced Industrial Science and Technology, Tokyo, 135-0064 Japan; 5 School of Data Sciences, Perdana University, 43400 Serdang, Malaysia; 6 Department of Chemistry and Biomolecular Sciences, Macquarie University, Sydney, NSW 2109 Australia

**Keywords:** InCoB, International conference on bioinformatics, APBioNet, Asia-Pacific bioinformatics network

## Abstract

The 16th International Conference on Bioinformatics (InCoB) was held at Tsinghua University, Shenzhen from September 20 to 22, 2017. The annual conference of the Asia-Pacific Bioinformatics Network featured six keynotes, two invited talks, a panel discussion on big data driven bioinformatics and precision medicine, and 66 oral presentations of accepted research articles or posters. Fifty-seven articles comprising a topic assortment of algorithms, biomolecular networks, cancer and disease informatics, drug-target interactions and drug efficacy, gene regulation and expression, imaging, immunoinformatics, metagenomics, next generation sequencing for genomics and transcriptomics, ontologies, post-translational modification, and structural bioinformatics are the subject of this editorial for the InCoB2017 supplement issues in BMC Genomics, BMC Bioinformatics, BMC Systems Biology and BMC Medical Genomics. New Delhi will be the location of InCoB2018, scheduled for September 26–28, 2018.

**Electronic supplementary material:**

The online version of this article (10.1186/s12864-017-4326-x) contains supplementary material, which is available to authorized users.

## Introduction

InCoB2017 was co-organized by The University of Technology Sydney, Tsinghua University, Graduate School at Shenzhen and APBioNet [1]. Six keynote and two invited talks covered the latest bioinformatics applications and big data developments in basic and applied biomedical research. The theme of big data-driven bioinformatics and precision medicine was highlighted in a panel discussion on its current status and future. Jianzhu Chen (Koch Institute for Integrative Cancer Research, Massachusetts Institute of Technology) presented bioinformatics-driven immunological research towards the identification of transcription factors in memory CD8^+^ T cell development, and the screening of bioactive and natural compounds that are able to induce human macrophages into either inflammatory or anti-inflammatory states. Yuelong Shu of the School of Public Health (Shenzhen) at Sun Yat-sen University and WHO Collaborating Center for Reference and Research on Influenza demonstrated how large-scale sequencing of human influenza virus in combination with antigenic surveillance of hemagglutinin using the computational platform PREDAC improved vaccine strain recommendations for China.

Single cell RNA sequencing can reveal small differences among cells which are important to know in understanding of cellular responses to signals and variations among one cell type. Yong Hou (BGI-Shenzhen) gave in his invited talk a comprehensive overview of single cell sequencing, its application in cancer research and potential to improve cancer diagnosis. Limsoon Wong (National University of Singapore) provided insight into reproducibility and coverage issues of mass spectrometry-based proteomics data, and introduced algorithms that produce more robust and biologically meaningful proteomic profiling results.

Two keynotes covered epigenetic modifications in embryonic stem cells from the perspectives of miRNA regulation and networks of chromatin-related proteins. Xiujie Wang (Institute of Genetics and Developmental Biology, Chinese Academy of Sciences) reported on clusters of miRNAs that were positively correlated with the pluripotency level of embryonic stem cells. One of the miRNAs was involved in a new form of mRNA regulation through N^6^-methyladenosine modification. Alfonso Valencia (Barcelona Supercomputing Center) concentrated in his talk on network-based approaches in epigenomics, evolution and biomedicine on the role of 5-hydroxymethylcytosine as a communication hub in the chromatin network of embryonic stem cells, and concluded with a network property analysis that revealed inverse as well as direct co-morbidities between Alzheimer’s disease, glioblastoma and lung cancer.

Saman Halgamuge (The Australian National University) and Mindy Shi (University of North Carolina at Charlotte) offered in their presentations an impressive demonstration of deep learning applications. Saman Halgamuge showed in his keynote successful applications of unsupervised deep learning in the areas of direct drug-brain interactions, drug repositioning and multi-electrode array workflow applications for screening pharmacological compounds. Mindy Shi utilized deep learning to construct predictive models for quantitative trait locus network analysis using genomic and interactome data.

The Annual General Meeting of APBioNet on September 20th was opened with the President’s Report. Among the reporting items was a new simplified membership fee structure with details available at APBioNet website [2], and plans to utilize BioRxiv preprint server [3] and its feature to transfer manuscripts to partnering journals for article submissions related to InCoB or InSyB (International Symposium of Bioinformatics). The winner of the bid for InCoB2018, Shandar Ahmad, introduced next year’s conference venue at Jawaharlal Nehru University, New Delhi [4]. Jim Hogan (Queensland University of Technology) presented an Expression of Interest (EOI) to host InCoB2019 or InCoB2020 at Gold Coast, Australia. Parties interested in hosting InCoB or InSyB as stand-alone, joint or back-to-back events are encouraged to submit an EOI through APBioNet’s website [5].

## Manuscript submission and review

In total 152 manuscripts were submitted through EasyChair conference management system [6] for consideration for publication as InCoB2017 supplement articles in Bioinformatics, BMC Genomics, BMC Bioinformatics, BMC Systems Biology, BMC Medical Genomics, IEEE/ACM Transactions on Computational Biology and Bioinformatics (TCBB), Journal of Bioinformatics and Computational Biology (JBCB) or PeerJ. After peer review by at least two reviewers of the Program Committee comprising 121 members, supported by 27 external sub-reviewers (Additional file 1), 65 (42.7%) manuscripts were provisionally accepted in revised form before the conference, pending final editorial approval. Fifty-seven articles are published in InCoB2017 supplement issues of BMC Bioinformatics (22) BMC Medical Genomics (7), BMC Systems Biology (14) and BMC Genomics (14). Eight articles will appear in PeerJ (1), JBCB (3), TCBB (3) and Bioinformatics (1). Best Paper Awards in the categories Gold, Silver and Bronze were given to authors of 28 manuscripts (Additional file 2). The articles included in the four BMC supplement issues are briefly summarized in Table 1 according to 12 topic groups arranged in alphabetical order.

**Table 1:** Summary of articles arranged by topic groups

Topic	Key finding or features
Algorithms	Divisive hierarchical maximum likelihood clustering (DRAGON), reduced search complexity O(n^2^c) [8].
	2D–EM two-step clustering approach applied to transcriptome and methylome data; filtering produces a feature matrix which is used as input for clustering by modified EM [9].
	Deep learning; convolutional neural network (CNN) using gradient boosted feature selection for classification of β-lactamases [10].
Bio-molecular networks	Prediction of protein complexes from protein-protein interaction networks utilizing gene expression data and protein functional annotations; CPredictor3.0 [11].
	Boolean network model simulation of signaling networks; changes of modularity and robustness by edge-removal mutations [12].
	Integrated protein-protein interaction network construction using DIP, Biogrid, Reactome and HPRD data; refinement/correction using relationship of functional similarity and proximity scores [13].
	Boolean network modeling; topology comparison of gene-gene dynamics influence and gene-gene molecular interaction networks [14].
	Integration of gene regulatory network inference with constraint-based metabolic models to simulate growth phenotype and exchange fluxes [15].
	Causal relationship detection between gene pairs for short time-series gene expression data (*E. coli*; yeast) based on lagged-coordinate delay embedding theorem [16].
	Cell fate predictions derived from polynomial modeling of human pancreatic single-cell gene expression data [17].
	Case study of network bi-stability and positive/negative feedback loops in TGF-β1 activation [18].
Cancer and disease informatics	Multi-view clustering method with enhanced consensus; breast cancer sub-typing and survival analysis [19].
	Network hubs as prognostic signatures in ovarian cancer, breast cancer and glioblastoma multiforme selected by Cox regression for correlating DNA methylation levels with outcome [20].
	Analysis pipeline in Python to classify tumors using a supervised machine-learning algorithm that predicts mutation status based on transcriptional patterns [21].
	Breast cancer outcome predictions from microarray data using Hadamard kernel [22].
	Application of node-weighted Steiner tree approach to identify proteins and protein-protein interactions in PI3K/Akt and MAPK signaling pathways; subnetwork identification [23].
	Tensor decomposition-based unsupervised feature extraction from gene expression data infers genes that induce post-traumatic stress disorder-mediated heart diseases and potential therapeutic targets [24].
	Graph regression-based approach which creates a unified framework for predicting binary, discrete and continued lncRNA-disease associations [25].
	Network consistency projection for human microbe-disease association predictions assuming that microbes with similar functions may have similar associated/not associated patterns with similar diseases [26].
	Target control problem with objectives-guided optimization algorithm to identify drivers (e.g. drug target nodes or network biomarkers) controlling targets in disease-associated networks [27].
Drug-target interactions and drug efficacy	Ligand-based quantitative structure-activity relationship modeling using Random Forest for drug target identification; web server application [28].
	Whole-body physiologically based pharmacokinetic modelling using constraint-based perturbation analysis with cluster Newton method; can handle mixed patient-dependent and patient-independent parameters [29].
	Ternary status based integer linear programming approach for MCF7 breast cancer cell line specific pathway network reconstruction and prediction of treatment efficacy of compounds using prior knowledge of literature and phosphoproteomic data [30].
	Core pharmacophore anchor model screening of FDA drugs to identify candidate dengue virus NS3 protease inhibitors [31].
	Dependency-based deep neural network model for drug-drug interaction feature extraction form Drug Bank [32].
Gene expression and regulation	Prediction of human transcriptional target genes using open chromatin regions, ChIP-seq data and transcription factor binding sites [33].
	Identification of phased secondary small interfering RNAs and miRNAs targeting PHAS loci in *Panax notoginseng* [34].
	Identification candidate tissue-specific circRNAs using bi-clustering of RNA-Seq-derived expression profiles [35].
	Multi-factor analysis of differential co-expression of breast cancer microarray data; identified differentially co-expressed sets containing *ESR1* and *CXCL13* [36]*.*
	Random Forest approach that uses motif combinations in prediction of cell-type-specific transcription factor binding sites [37].
Imaging	Discriminant filter bank approach for extracting EEG signal features using common spatial patterns; low misclassification rate [38].
	MatQuantify is a software for assessment of fluorescence microscopy images of mitotic spindles and their architecture changes [39].
	Phytoplankton and zooplankton image classification using a non-linear multiple kernel learning approach [40].
Immuno-informatics	Computational methodology pipeline to process, predict and analyze potential T cell epitopes using influenza A, dengue, West Nile hepatitis A and HIV-1 virus sequence data [41].
	NetCTL-bases predictions of HLA-A2, -A3 and -B7 supertype-restricted Zaire ebolavirus T cell epitope canidates [42].
	Agent based-model to simulate citrus-derived flavones as vaccine adjuvants against human papilloma virus 16; mouse in vivo result confirmation [43].
	Investigation of differences in cellular A-to-I RNA editing activities upon infection with influenza A virus H1N1 and H3N2 [44].
	RNA-Seq based analysis of differential innate immune response of RNA-Seq human cells infected with H1N1,H3N1, H5N1, HALo mutant and H7N9 and chicken and quail cells infected with H5N1 and H5N2 [45].
	Cellular RNA editing analysis of *C. albicans* infected human epithelial cell lines and mouse in vivo infected tongue and kidney tissues [46].
Meta-genomics	16sPIP analysis pipeline for classification of 16S rDNA NGS data and screening of 346 clinically relevant pathogens [47].
	CoMet binning workflow was used to assess contig coverage in combination with GC content for automated binning of a single and multi-strain metagenomic samples [48].
	Metagenomic and -transcriptomic analysis of oolong teas to identify dominant microbial species and their anti-microbial peptides [49].
	Computational pipeline ezTree infers marker genes and maximum likelihood phylogenetic trees from uncultivated prokaryotic genomes [50].
NGS genomics and transcript-omics	GTZ is a fast and lossless compression tool for cloud computing of FASTQ files; data transmission can overlap with compression; [51].
	Coverage-dependent (from RNA sample concentration) RNA-Seq approach using a Bayesian method that infers the posterior distribution of a true gene count [52].
	GT-WGS is a distributed whole-genome computing system based on Amazon Web Service cloud computing platform [53].
	Pan-genome tool PGAP-X is a cross-platform software to analyze and visualize genome structure dynamics and and gene content [54].
	Pará rubber (*H. brasiliensis*) genome transcriptome database [55].
Ontologies	InfAcrOnt tool can calculate similarities between terms across different ontologies and support the identifcation of novel relationships [56].
	CroGO2 is an iterative ranking-based method that measures similarities of cross-categories GO terms using GO term level and interaction information in gene co-function networks [57].
	Ontology of Chinese Medicine for Rheumatism represents 26 anti-rheumatism Chinese drugs together with their chemical ingredients, adverse effects and related information [58].
Post-translational modification sites	MDD-Carb is a tool for prediction of carbonylation sites utilizes maximal dependence decomposition and profile hidden Markov models [59].
	SUCCESS is a SVM-based tool that predicts succinylation sites using structural and evolutionary information of amino acids [60].
	CruxPTM is a platform for structure-based analysis post-translational modifications in context of PPI and drug binding [61].
Structural bio-informatics	Molecular dynamics analysis of charge states of balanol analogues that are ATP competitive inhibitors but nonselective for protein kinases A and C [62].
	DeepSacon tool is a sparse autoencoder-based deep neural network to predict solvent accessibility and contact numbers [63].
	R3D-BLAST2 is an improved search tool for similar RNA 3D substructures that can handle mmCIF files [64].

## Conclusion

The potpourri of bioinformatics research output showcased at InCoB2017 reflects APBioNet’s goal to cater to a diverse range of practitioners and developers in the field. One of the highly cited articles of the InCoB conference series is an evaluation of human protein-protein interaction data in the public domain by Mathivanan et al. [7] with an average of ten citations per year. The paper was presented at InCoB2006 in New Delhi where next year’s conference will be held.

## Additional files

Additional file 1: List of InCoB2017 Reviewers. (PDF 52 kb)

Additional file 2: InCoB2017 Best Paper Awards. (PDF 73 kb)
