# Supplementary material for: A bioinformatics potpourri
Source: BMC Genomics. 2018 Jan 19;19(Suppl 1):920. doi: 10.1186/s12864-017-4326-x (PMC5780851; doi:10.1186/s12864-017-4326-x)
Supplement: Supplementary file 2 — InCoB2017 Best Paper Awards. (PDF 73 kb) [file 12864_2017_4326_MOESM2_ESM.pdf]

Additional File 2. InCoB2017 Best Paper Awards

Category	Journal supplement
	**BMC Genomics**
	Wu Y-W. **ezTree: an automated pipeline for identifying phylogenetic marker genes and inferring evolutionary relationships among uncultivated prokaryotic draft genomes**. *BMC Genomics*. 2017; **18** Suppl 11:S1.<br>Lim WC and Khan AM. **Mapping HLA-A2, A3 and B7 supertype-restricted T-cell epitopes in the ebolavirus proteome**. *BMC Genomics*. 2017; **18** Suppl 11:S2.<br>Huang Y, Cao Y, Li J, Liu Y, Zhong W, Li X, Chen C, Hao P. **A survey on cellular RNA editing activity in response to *Candida albicans* infections**. *BMC Genomics*. 2017; **18** Suppl 11:S3.<br>Cao Y, Cao R, Huang Y, Zhou H, Liu Y, Li X, Zhong W, Hao P. **A comprehensive study on cellular RNA editing activity in response to infections with different subtypes of influenza A viruses**. *BMC Genomics*. 2017; **18** Suppl 11:S4.
**Gold**<br>(11)	**BMC Medical Genomics**
	Khan AM, Hu Y, Miotto O, Thevasagayam NM, Sukumaran R, Raman HSA, Brusic V, Tan TW, August JT. **Analysis of viral diversity for vaccine target discovery**. *BMC Medical Genomics*. 2017; **10** Suppl 4:S1.
	**BMC Bioinformatics**
	Zheng H, Wang R, Yu Z, Wang N, Gu Z, Zheng B. **Automatic plankton image classification combining multiple view features via multiple kernel learning**. *BMC Bioinformatics*. 2017; **18** Suppl 16:S1.<br>Hardianto A, Yusuf M, Liu F, Ranganathan S. **Exploration of charge states of balanol analogues acting as ATP-competitive inhibitors in kinases**. *BMC Bioinformatics*. 2017; **18** Suppl 16:S2.

	Yen C-Y, Lin J-C, Chen K-T, Lu CL. **R3D-BLAST2: an improved search tool for similar RNA 3D substructures**. *BMC Bioinformatics*. 2017; **18** Suppl 16S3.
	**BMC Systems Biology**
	Ji Z, Wang B, Yan K, Dong L, Meng G, Shi L. **A linear programming computational framework integrates phosphor-proteomics and prior knowledge to predict drug efficacy**. *BMC Systems Biol*. 2017; **11** Suppl 7:S1.<br>Ma L and Jie Zheng J. **A polynomial based model for cell fate prediction in human diseases**. *BMC Systems Biol*. 2017; **11** Suppl 7:S2.<br>Huang K-Y, Chang T-Z, Jhong J-H, Chi Y-H, Li W-C, Chan C-L, Lai KR, Lee T-Y. **Identification of natural antimicrobial peptides from bacteria through metagenomic and metatranscriptomic analysis of high-throughput transcriptome data of Taiwanese oolong teas**. *BMC Systems Biol*. 2017; **11** Suppl 7:S3.
**Silver** (4)	**BMC Genomics**
	Chen K, Liu L, Zhang X, Yuan Y, Ren S, Guo J, Wang Q, Liao P, Li S, Cui X, Li Y-F, Zheng Y. **Phased secondary small interfering RNAs in *Panax notoginseng***. *BMC Genomics*. 2017; **18** Suppl 11:S5.
	**BMC Medical Genomics**
	Taguchi Y-H. **Tensor decomposition-based unsupervised feature extraction identifies candidate genes that induce post-traumatic stress disorder-mediated heart diseases**. *BMC Medical Genomics*. 2017; **10** Suppl 4:S2.
	**BMC Bioinformatics**
	Peng J, Wang H, Lu J, Hui W, Wang Y, Shang X. **Identifying term relations cross different gene ontology categories**. *BMC Bioinformatics*. 2017; **18** Suppl 16:S6.
	**BMC Systems Biology**
	Xu Y, Zhou J, Zhou S, Guan J. **CPredictor3.0: Effectively detecting protein complexes**

	**from PPI networks with expression data and functional annotations**. *BMC Systems Biol*. 2017; **11** Suppl 7:S4.
**Bronze** (14)	**BMC Genomics**
	Weifeng G, Shaowu Z, Qianqian S, Chengming Z, Tao Z, Luonan C. **A novel algorithm for finding optimal driver nodes to target control complex networks and its applications for drug targets identification**. *BMC Genomics*. 2017; **18** Suppl 11:S6.<br>Makita Y, Kawashima M, Lau NS, Matsui M, Othman AS. **Construction of Pará rubber tree genome and multi-transcriptome database accelerates rubber researches**. *BMC Genomics* 2017; **18** Suppl 11:S7.
	**BMC Medical Genomics**
	Dayton J and Piccolo S. **Classifying cancer genome aberrations by their mutually exclusive effects on transcription**. *BMC Medical Genomics*. 2017; **10** Suppl 4:S3.
	**BMC Bioinformatics**
	Miao J, Han N, Qiang Y, Zhang T, Li X, Zhang W. **16sPIP: a comprehensive analysis pipeline for rapid pathogen detection in clinical samples based on 16S metagenomic sequencing**. *BMC Bioinformatics*. 2017; **18** Suppl 16:S22.<br>Pathak N, Lai M-L, Chen W-Y, Hsieh B-W, Yu G-Y, Yang J-M. **Pharmacophore anchor models of flaviviral NS3 proteases lead to drug repurposing for DENV infection**. *BMC Bioinformatics*. 2017; **18** Suppl 16:S4.<br>Lee K, Lee M, Kim D. **Utilizing Random Forest QSAR models with optimized parameters for target identification and its application to target-fishing server**. *BMC Bioinformatics*. 2017; **18** Suppl 16:S7.<br>Sun Y, Ma C, Halgamuge S. **The node-weighted Steiner tree approach to identify elements of cancer-related signaling pathways**. *BMC Bioinformatics*. 2017; **18** Suppl 16:S5.

	**BMC Systems Biology**
	Su M-G, Weng JT-Y, Hsu JB-K, Huang K-H, Chi Y-H, Lee TY. **Investigation and identification of functional post-translational modification sites associated with drug binding and protein-protein interactions**. *BMC Systems Biol*. 2017; **11** Suppl 7:S6.<br>Banos DT, Elati M, Trébulle P. **Integrating transcriptional activity in genome-scale models of metabolism**. *BMC Systems Biol*. 2017; **11** Suppl 7:S7.<br>Li H, Venkatraman L, Narmada BC, White J, Yu H, Tucker-Kellogg L. **Computational analysis reveals the coupling between bistability and the sign of a feedback loop in a TGF-β1 activation model**. *BMC Systems Biol*. 2017; **11** Suppl 7:S8.<br>Mazaya M, Trinh H-C, Kwon Y-K. **Construction and analysis of gene-gene dynamics influence networks based on a Boolean model**. *BMC Systems Biol*. 2017; **11** Suppl 7:S9.<br>Truong CD and Kwon Y-K. **Investigation on changes of modularity and robustness by edge-removal mutations in signaling networks**. *BMC Systems Biol*. 2017; **11** Suppl 7:S5.<br>Liu Q, Wang J, Zhu Y, He Y. **Ontology-based systematic representation and analysis of traditional Chinese drugs against rheumatism**. *BMC Systems Biol*. 2017; **11** Suppl 7:S10.<br>Kao H-J, Weng S-L, Huang K-Y, Kaunang F-J, Hsu, JB-K, C-H, Huang C-H, Lee T-Y. **MDD-Carb: a combinatorial model for the identification of protein carbonylation sites with substrate motifs**. *BMC Systems Biol*. 2017; **11** Suppl 7:S11.
