# Supplementary material for: A bioinformatics potpourri
Source: BMC Genomics. 2018 Jan 19;19(Suppl 1):920. doi: 10.1186/s12864-017-4326-x (PMC5780851; doi:10.1186/s12864-017-4326-x)
Supplement: Supplementary file 1 — List of InCoB2017 Reviewers. (PDF 52 kb) [file 12864_2017_4326_MOESM1_ESM.pdf]

Additional File 1. List of InCoB2017 Reviewers

We thank the reviewers who served on the InCoB2017 Scientific Program Committee and the sub-reviewers for completing reviews of manuscripts submitted to the InCoB2017 supplement issues of BMC Genomics, BMC Medical Genomics, BMC Bioinformatics, BMC Systems Biology, Journal of Bioinformatics and Computational Biology, or IEEE/ACM Transactions on Computational Biology and Bioinformatics.

InCoB2017 Scientific Program Committee:

1. Catherine Abbott, Flinders University, Australia
2. Shandar Ahmad, Jawaharlal Nehru University, India
3. Tatsuya Akutsu, Kyoto University, Japan
4. Masanori Arita, National Institute of Genetics, Japan
5. Nicola Armstrong, Murdoch University, Australia
6. LamiaeAzizi, The University of Sydney, Australia
7. Vladimir Bajic, King Abddulah University of Science and Technology, Saudi Arabia
8. Abdul Baten, Southern Cross University, Australia
9. Michael A. Beer, Johns Hopkins University, USA
10. Mikael Boden, The University of Queensland, Australia
11. Peter Bond, A*STAR, Singapore
12. Vladimir Brusic, Griffith University, Australia
13. Filippo Castiglione, National Research Council of Italy, Italy
14. Jonathan Chan, King Mongkut's University of Technology Thonburi, Thailand
15. Michael Charleston, The University of Sydney, Australia
16. Peng Chen, Anhui University, P.R. China
17. Ming Chen, Zhejiang University, P.R. China

18. Brian Chen, Lehigh University, USA
19. Wai-KiChing, The University of Hong Kong, Hong Kong S.A.R.
20. Melissa Davis, Walter and Eliza Hall Institute of Medical Research, Australia
21. Mahmoud Elhefnawi, American University in Cairo, Egypt
22. Mohd Firdaus-Raih, Universiti Kebangsaan Malaysia, Malaysia
23. Ge Gao, Peking University, P.R. China
24. Lin Gao, Xidian University, P.R. China
25. Susumu Goto, Research Organization of Information and Systems
26. Michael Gromiha, Indian Institute of Technology Madras, India
27. Yongqun He, University of Michigan, USA
28. Matthew He, Nova Southeastern University, USA
29. Barbara Holland, University of Tasmania, Australia
30. Wing-Kai Hon, Tsinghua University, P.R. China
31. Paul Horton, National Institute of Advanced Industrial Science & Technology, Japan
32. Fang Hsu, Feng Chia University, Taiwan
33. Chia-Lang Hsu, National Taiwan University Hospital, Taiwan
34. Wenlian Hsu, Academia Sinica, Taiwan
35. Guang Hu, Soochow University, P.R. China
36. Chun-His Huang, University of Connecticut, USA
37. Ming-Jing, Academia Sinica, Taiwan
38. Ulykbek Kairov, Nazarbayev University, Kazakhstan
39. Paul Kennedy, University of Technology Sydney, Australia
40. Asif M. Khan, Perdana University, Malaysia
41. Tsung Fei Khang, University of Malaya, Malaysia
42. Daisuke Kiga, Waseda University, Japan

43. Akihiko Konagaya, Tokyo Institute of Technology, Japan
44. Shinji Kondo, National Institute of Polar Research, Japan
45. Anton Kratz, RIKEN, Japan
46. Igor Kurochkin, Sysmex Corp., Japan
47. Chee Keong Kwoh, Nanyang Technological University, Singapore
48. Yinglei Lai, George Washington University, USA
49. Ruiting Lan, The University of New South Wales, Australia
50. Thuc Duy Le, University of South Australia, Australia
51. Chih Lee, Illumina Inc., USA
52. Jie Li, Harbin Institute of Technology, P.R. China
53. Jinyan Li, University of Technology Sydney, Australia
54. Haiquan Li, University of Arizona, USA
55. Wentian Li, Feinstein Institute for Medical Research, USA
56. Xiaoli Li, A*STAR, Singapore
57. Zhenhua Li, National University of Singapore, Singapore
58. Lin Liu, University of South Australia, Australia
59. Tao Liu, The University of New South Wales, Australia
60. Yuansheng Liu, University of Technology Sydney, Australia
61. Xiaoke Ma, Xidian University, P.R. China
62. Sorayya Malek, University of Malaya, Malaysia
63. Hiroshi Mamitsuka, Kyoto University, Japan
64. Prashanti Manda, University of North Carolina, USA
65. Jessica Mar, The University of Queensland, Australia
66. Osamu Maruyama, Kyushu University, Japan
67. Hideo Matsuda, Osaka University, Japan

68. Lenny Moise, University of Rhode Island, USA
69. Shinichi Morishita, The University of Tokyo, Japan
70. Santo Motta, University of Catania, Italy
71. Hampapathalu Nagarajaram, University of Hyderabad, India
72. Kenta Nakai, The University of Tokyo, Japan
73. Ka-Lok Ng, Asia University, Taiwan
74. Francesco Pappalardo, University of Catania, Italy
75. Ashwini Patil, Institute of Medical Science, The University of Tokyo, Japan
76. Shaoliang Peng, National University of Defense Technology, P.R. China
77. Jiajie Peng, Northwestern Polytechnical University, P.R. China
78. Nikolai Petrovsky, Flinders Medical Centre, Australia
79. Gajendra Raghava, Institute of Microbial Technology, India
80. Shoba Ranganathan, Macquarie University, Australia
81. Yasubumi Sakakibara, Keio University, Japan
82. Daniele Santoni, National Research Council of Italy, Italy
83. Christian Schönbach, Kumamoto University, Japan
84. Masakazu Sekijima, The University of Tokyo, Japan
85. Hongbin Shen, Shanghai Jiaotong University, P.R. China
86. Tetsuo Shibuya, The University of Tokyo, Japan
87. Muhammad Farhan Sjaugi, Perdana University, Malaysia
88. Narayanaswamy Srinivasan, Indian Institute of Science, India
89. Ran Su, Tianjin University, P.R. China
90. Chinh Tran-To Su, Nanyang Technological University, Singapore
91. Xiaoming Sun, Harvard University, USA
92. Jianqiang Sun, National Institute of Advanced Industrial Science &Technology, Japan

93. Yanni Sun, Michigan State University, USA
94. Sing-Hoi Sze, Texas A&M University, USA
95. Y-H. Taguchi, Chuo University, Japan
96. Yoichi Takenaka, Kansai University, Japan
97. Martti Tammi, National University of Singapore, Singapore
98. Paolo Tieri, National Research Council of Italy, Italy
99. Ikuo Uchiyama, National Institute for Basic Biology, Japan
100. Mauno Vihinen, Lund University, Sweden
101. Dongqing Wei, Shanghai Jiaotong University, P.R. China
102. Thomas Wong, The Australian National University, Australia
103. Limsoon Wong, National University of Singapore, Singapore
104. Qingyao Wu, South China University of Technology, P.R. China
105. Yufeng Wu, University of Connecticut, USA
106. Juanying Xie, Shaanxi Normal University, P.R. China
107. Yingqiu Xie, Nazarbayev University, Kazakhstan
108. Yee Hwa Yang, The University of Sydney, Australia
109. Ueng-Cheng Yang, National Yang Ming University, Taiwan
110. Kevin Yip, The Chinese University of Hong Kong, Hong Kong S.A.R.
111. Siu Ming Yiu, The University of Hong Kong, Hong Kong S.A.R.
112. Zuguo Yu, Xiangtan University, P.R. China
113. Tao Zeng, Nanyang Technological University, Singapore
114. Guang Lan Zhang, Boston University, USA
115. Liang Zhao, Hubei University of Medicine, P.R. China
116. Xing-Ming Zhao, Tongji University, P.R. China
117. Zhongming Zhao, University of Texas Health Science Center at Houston, USA

118. Yun Zheng, Fudan University, P.R. China
119. Shuigeng Zhou, Fudan University, P.R. China
120. Yaoqi Zhou, Griffith University, P.R. China
121. Shanfeng Zhu, Fudan University, P.R. China

Sub-reviewers (27): Ibrahim Alabdulmohsin, Henrique Assis Lopes Ribeiro, Wang Bingbo, Ching-Tai Chen, Stefano Elli, Epifanio Fichera, Milana Frenkel-Morgenstern, Jianzheng Huang, Songwei Jia, Simon Lee, Xin Li, Kuo-Ching Liang, Xiaoke Ma, Enrico Mastrostefano, Alyaa Mohamed, Olaa Motwalli, Yutaka Saito, Marnie Shaw, Yu-Shuen Tsai, Ziye Wang, Taosheng Xu, Shuwei Yao, Yusen Ye, Ronghui You, Chin-Sheng Yu, Junpeng Zhang, Shengli Zhang.
